# Association between Cognitive Impairment Severity and Polypharmacy in Older Patients with Atrial Fibrillation: A Retrospective Study Using Inpatient Data from a Specialised Geriatric Hospital

**DOI:** 10.3390/geriatrics9010015

**Published:** 2024-01-24

**Authors:** Yoshitomo Shimazaki, Keiko Kishimoto, Joji Ishikawa, Rika Iwakiri, Atsushi Araki, Shinobu Imai

**Affiliations:** 1 Division of Pharmacy, Tokyo Metropolitan Institute for Geriatrics and Gerontology, 35-2 Sakae-cho, Itabashi-ku, Tokyo 173-0015, Japan; 2 Depertment of Pharmacoepidemiology, Showa University Graduate School of Pharmacy, 1-8-5, Hatanodai, Shinagawaku, Tokyo 142-8555, Japan; 3 Department of Social Pharmacy, Showa University Graduate School of Pharmacy, 1-8-5, Hatanodai, Shinagawaku, Tokyo 142-8555, Japan; kishimoto-k@pharm.showa-u.ac.jp; 4 Division of Cardiology, Tokyo Metropolitan Institute for Geriatrics and Gerontology, 35-2 Sakae-cho, Itabashi-ku, Tokyo 173-0015, Japan; 5 Division of Elderly Care, Tokyo Metropolitan Institute for Geriatrics and Gerontology, 35-2 Sakae-cho, Itabashi-ku, Tokyo 173-0015, Japan; 6 Frail Prevention Center, Training Center, Tokyo Metropolitan Institute for Geriatrics and Gerontology, 35-2 Sakae-cho, Itabashi-ku, Tokyo 173-0015, Japan; aaraki@tmghig.jp

**Keywords:** atrial fibrillation, cognitive function, comprehensive geriatric assessment, Dementia Assessment Sheet for Community-based Integrated Care System 21-Items, polypharmacy

## Abstract

This study aimed to investigate the association between cognitive impairment and polypharmacy in patients with atrial fibrillation prone to cognitive decline, and to elucidate if the Dementia Assessment Sheet for Community-based Integrated Care System 21-Items (DASC-21) severity classification indicates drug adjustment. This retrospective cohort study used the DASC-21 and Diagnosis Procedure Combination data at a specialised geriatric hospital with patients hospitalised between April 2019 and March 2022. The association between cognitive severity evaluated using the DASC-21 and polypharmacy was investigated using a multivariate logistic regression model. Data of 1191 inpatients (44.3% aged ≥85 years, 49.0% male) were analysed. Compared with severe cognitive impairment, mild (odds ratio [OR]: 3.33, 95% confidence interval [CI]: 1.29–8.57) and moderate (OR: 2.46, 95% CI: 1.06–5.72) impairments were associated with concurrent use of ≥6 medications. Antithrombotics were related to polypharmacy. The ORs did not change with 6, 8, or 10 medications (2.11 [95% CI: 1.51–2.95, *p* < 0.001], 2.42 [95% CI: 1.79–3.27, *p* < 0.001], and 2.01 [95% CI: 1.46–2.77, *p* < 0.001], respectively). DASC-21 severity was associated with polypharmacy in patients with atrial fibrillation, with a trend toward decreased polypharmacy from moderate to severe. The DASC-21 may serve as an indicator for drug adjustment in clinical practice.

## 1. Introduction

Atrial fibrillation is a disease whose severity increases with age, and its global prevalence increases yearly. A 10-year analysis of the Framingham Heart Study revealed high rates of the incidence, prevalence, and risk factors of atrial fibrillation, such as post-onset stroke and mortality [1]. The prevalence of atrial fibrillation increases approximately four-fold in men and women. An age-adjusted incidence rate also shows a similar trend [1]. Since atrial fibrillation is a high risk factor for cerebral infarction, antithrombotic medications are the mainstay of medication therapy to prevent cerebral infarction.

Older patients often have multimorbidity, difficulties in diet and exercise therapy, and poor medication adherence; therefore, polypharmacy is common among them. Polypharmacy is associated with increased rates of adverse events, including drug interactions, adverse drug events (ADEs), falls due to dizziness, hospitalisation, prolonged hospital stays, readmission immediately after discharge, and mortality [2,3,4]. In a meta-analysis, the pooled prevalence of adverse drug reactions in older inpatients was 22%, and polypharmacy and potentially inappropriate medication use were predictors of ADEs during hospitalisation [5]. In line with this, various methods for improving polypharmacy have been assessed, including the development of qualitative assessment tools such as the Screening Tool of Older Persons’ Prescriptions (STOPP) criteria [6] and Beers criteria [7], as well as proposals to understand the reasons for taking medications and assessing medication risks and benefits [8]. Reducing or modifying inappropriate prescriptions has been validated in randomised trials [9,10,11] and observational studies [12,13], thereby recognising the effectiveness of pharmacist-led interventions.

Cognitive decline has also been reported to be closely associated with medication adherence [14,15,16,17] and may be an essential indicator for clinicians to decide whether or not to continue medication therapy. Regarding medication adjustment, which includes deprescribing as a process to improve medication adherence in the older patients, the suitability of the Comprehensive Geriatric Assessment (CGA) [18,19], a method that represents problems in areas such as cognitive function, activities of daily living (ADLs), psychology, nutrition, medications, and social status of patients, has been reported [20,21,22,23,24]. The Dementia Assessment Sheet for Community-based Integrated Care System 21-Items (DASC-21) is an effective tool for CGA. It can be used to evaluate behavioural changes related to cognitive impairment and impairment in daily living. It is characterised by its full range of instrumental activities of daily living (IADLs; six items), making it easy to detect impairment in daily living among individuals with mild dementia [25]. In this regard, the DASC-21 may be more likely to identify executive functions, including self-management of medications, than other cognitive function screenings, and is considered reliable, especially when completed by family members and caregivers who know the patient well [25]. The higher the DASC-21 score, the more severe the cognitive impairment and the likelier it is the time to consider medication adjustments for older patients with advanced functional disability. Patients with atrial fibrillation are prone to multimorbidity and polypharmacy due to rate control. Medication adjustments are often required to avoid adverse events because polypharmacy in atrial fibrillation is associated with increased breeding and all-cause mortality [26]. However, it is unclear how cognitive function affects polypharmacy in older patients with atrial fibrillation.

Therefore, this study aimed to investigate the association between cognitive impairment and polypharmacy in patients with atrial fibrillation to elucidate an indicator for medication adjustment by professionals in health care.

## 2. Methods

### 2.1. Study Design and Data Sources

This retrospective, cross-sectional study used the DASC-21 with the Diagnosis Procedure Combination (DPC) data [27] at the Tokyo Metropolitan Institute for Geriatrics and Gerontology, an acute-care hospital for older patients. Since its establishment, this hospital has been conducting interdisciplinary research on ageing as a core institute in Japan. It functions as a knowledge bank and a source for capable researchers in gerontology. In our study, the data of patients hospitalised and discharged between April 2019 and March 2022 were used. DPC data have been collected in Japan since 2003 as a component of the case-mix system implemented in acute-care hospitals [28]. The DASC-21 questionnaire reflects cognitive and life functions [25] and is frequently used in geriatric hospitals.

### 2.2. Study Population

Data on hospitalised patients diagnosed with atrial fibrillation following the International Classification of Diseases, 10th Revision (ICD-10) codes I480, I481, I482, and I489 were extracted from the DPC database and disease names at discharge. We extracted data on patients with atrial fibrillation comorbidities that could not be identified in the DPC data to extract more patients with atrial fibrillation. Furthermore, patient responses to the DASC-21 were identified. Patients without regular oral medications, missing co-payment and ADL information, and who died during hospitalisation were excluded.

### 2.3. Ethics Consideration

The ethics committee of the Tokyo Metropolitan Geriatric Hospital and Research Institute approved this study (approval no. R22-021). The opt-out consent model was adopted because the authors received all data after anonymisation. All analyses followed the tenets of the Declaration of Helsinki.

### 2.4. Variables

The outcome measurement was polypharmacy, defined as the concurrent use of ≥6 medications (the definition of polypharmacy in other countries is five or more medications, but this study was conducted only in one hospital in Japan, so the Japanese definition of six or more medications was used.). The thresholds were changed to ≥8 or ≥10 to assess the level of polypharmacy. Since prescriptions for primary medical care are determined the day before discharge, the number of medications (oral, patch, and inhalation medications) used a day before discharge was considered as the number of medications used regularly at home. DASC-21 responses provided by the patient’s family or caregiver were obtained before admission at the hospital [25]. If family members or caregivers were unavailable, the responses of the patient or a care manager were considered. A previous study reported that the Cronbach’s alpha coefficient values for the DASC-21 responses provided by patients’ family members, other responders, and trained nurses were 0.934, 0.950, and 0.808, respectively, and that the tool is sufficiently reliable and valid for assessing cognitive and life function impairment, detecting dementia, and assessing dementia severity [25]. The results obtained using the DASC-21 correlate significantly with those obtained using the Mini-Mental State Examination (MMSE), Frontal Assessment Battery, and Clinical Dementia Rating (CDR) total and box scores [25]. The DASC-21 data were classified into four categories based on the DASC-21 Assessment Manual: (1) normal (no cognitive impairment), (2) mild cognitive impairment, (3) moderate cognitive impairment, and (4) severe cognitive impairment [29]. Patients’ characteristics, such as sex, age, height, weight, patient co-payment information, ADLs at admission and discharge, length of hospital stay (LOS), diagnosis codes, emergency hospitalisation, discharge destination (household, transfer), hospitalisation pathway (household, transfer), and medications were extracted from the DPC data. Age was classified as ≤74 years, 75–84 years (the late stage of ageing in Japan), and ≥85 years. LOS was divided into interquartile ranges, and we used the fourth quartile, where hospitalisation is prolonged, as the variable. Furthermore, data on antithrombotic medications (direct oral anticoagulants and warfarin), benzodiazepines (BZs), proton pump inhibitors (PPIs), 3-hydroxy-3-methylglutaryl-coenzyme A reductase inhibitors (statins), and medications identified using the Screening Tool for Older Person’s Appropriate Prescriptions for Japanese (STOPP-J) [30] (Supplementary File S1), were extracted from the DPC data. BZs were excluded from the original STOPP-J. The Charlson Comorbidity Index (CCI) was calculated following the coding algorithms by Quan et al. and used as a measure of the chronic illness burden [31].

### 2.5. Statistical Analysis

To summarise patient characteristics, continuous variables are expressed as the mean and standard deviation or the median and interquartile range (IQR), depending on the variable distribution. Categorical variables are expressed as proportions. Differences between groups were compared using the Mann–Whitney *U* test or chi-squared test. The association between the severity of cognitive impairment, as assessed using the DASC-21, and polypharmacy was investigated using a multivariate logistic regression model adjusted for the following covariates: sex, age, body mass index (BMI), LOS, hospitalisation pathway, emergency admission, discharge destination, CCI, connective tissue disease/rheumatic disease, peptic ulcer disease, diabetes without complications, diabetes with complications, renal disease, antithrombotics, BZs, medications identified using STOPP-J, and statins. Adjusted odds ratios and 95% confidence intervals (CIs) were calculated. Multiple comparisons of the number of medications per 100 patients according to the four categories based on the DASC-21 were performed using Steel’s test and referenced severe classification. Statistical significance was set at a two-tailed *p*-value of ≤0.05. All analyses were conducted using JMP Pro 16.2.0 (SAS Institute Inc., Cary, NC, USA).

## 3. Results

**Figure 1:**
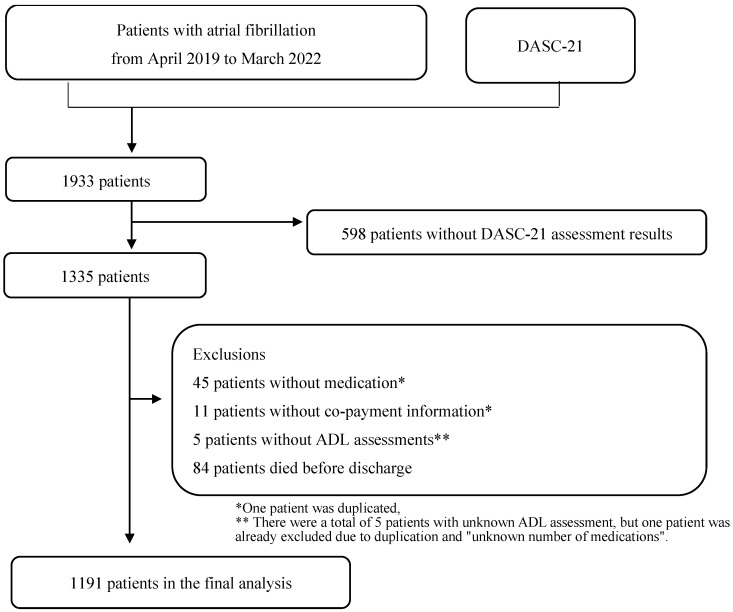
Flow diagram of patient selection.

**Figure 2:**
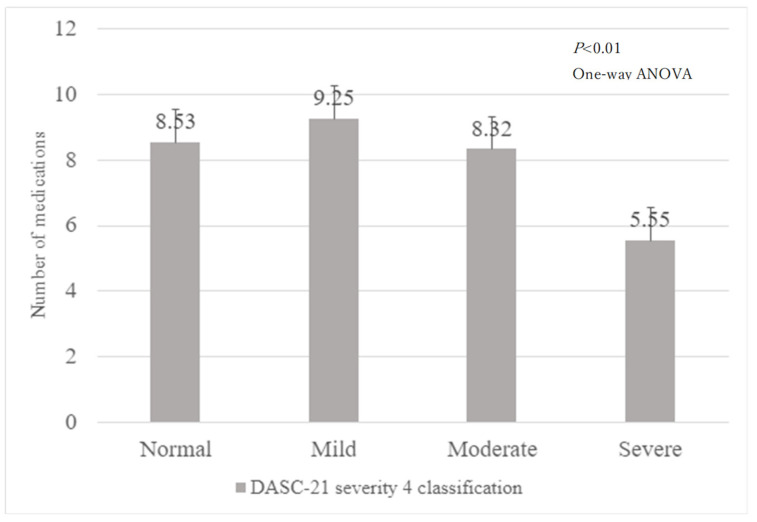
Number of medications by DASC-21 severity 4 classification.

Figure 1 shows the flowchart of patient selection. A total of 1933 inpatients participated in this study. However, after excluding 598 patients without the DASC-21 responses, 45 with unknown or no medications, 11 with missing co-payment information, 2 with unknown ADL assessment results at admission, 3 with unknown ADL assessment results at discharge, and 84 who died during hospitalisation, only 1191 patients were included in the final analysis. Figure 2 shows the relationship between the four DASC-21 severity categories and the number of medications.

DASC-21: Dementia Assessment Sheet for Community-based Integrated Care System 21-Items and ADL: activity of daily living.

Patient characteristics are summarised in Table 1. Of the 1191 patients, 15.6%, 40.1%, and 44.3% were aged ≤74 years, 75–84 years, and ≥85 years, respectively. Furthermore, 16.5%, 60.1%, and 23.3% had a BMI of <18.5 kg/m^2^, 18.5–25 kg/m^2^, and ≥25 kg/m^2^, respectively. The CCI was 0, 1 or 2, and 3 for 10.1%, 57.6%, and 32.3% of the patients, respectively. Medications identified using STOPP-J were used by 14.0% of the patients. The overall mean number of medications was 8.0 (IQR: 6–11), with 4.0 (IQR: 3–5) patients taking ≤5 medications, and 9.0 (IQR: 8–12) taking ≥6 medications. The mean DASC-21 score of all patients was 28.0 (IQR: 23–45).

Table 2 shows the association between cognitive impairment severity as determined based on the DASC-21 and polypharmacy. Compared with severe impairment, mild and moderate cognitive impairments were associated with using ≥6 medications (OR: 3.33, 95% CI: 1.29–8.57; OR: 2.46, 95% CI: 1.06–5.72, respectively). However, DASC-21 cognitive severity, normally referenced as severe, was not associated with the use of ≥6 polypharmacy medications (OR: 2.04, 95% CI: 0.84–4.94), but was associated with the use of ≥8 (OR: 3.13, 95% CI: 1.23–8.01) and ≥10 medications (OR: 3.76, 95% CI: 1.04–13.5). The patients who used ≥10 medications were older (OR: 1.55, 95% CI: 1.01–2.40), had a longer LOS (OR: 1.69, 95% CI: 1.21–2.37), and had many comorbidities (OR: 1.63, 95% CI: 0.95–2.81). Antithrombotic medication use was associated with polypharmacy, and the ORs did not change significantly regardless of whether the patients were taking >6, 8, or 10 medications (2.11 [95% CI: 1.51–2.95, *p* < 0.001], 2.42 [95% CI: 1.79–3.27, *p* < 0.001], and 2.01 [95% CI 1.46–2.77, *p* < 0.001], respectively).

Table 3 shows a multiple comparison analysis for the mean number of medications on each severity referenced as severe classification. Regarding the numbers of medication for antithrombotics (Normal: *p* = 0.501, Mild: *p* = 0.855, and Moderate: *p* = 0.855) and STOPP-J (Normal: *p* = 0.577, Mild: *p* = 0.491, and Moderate: *p* = 0.356), there was no decrease in the number of medications compared with severe classification. The number of medications in antithrombotics was stable among normal, mild, and moderate DASC-21 severity cognitive impairment compared with severe (76.7 ± 42.3, 73.4 ± 44.3, 73.2 ± 44.3, and 69.6 ± 46.7, respectively). A similar trend was observed for STOPP-J (8.9 ± 31.6, 19.6 ± 41.4, 21.7 ± 43.9, and 12.1 ± 33.1, respectively).

DASC-21: Dementia Assessment Sheet for Community-based Integrated Care System 21-Items; SD: standard deviation; and STOPP-J: Screening Tool for Older Person’s Appropriate Prescriptions for Japanese. 

## 4. Discussion

This study assessed the association between cognitive impairment and polypharmacy in patients with atrial fibrillation to determine the indicators of medication adjustment by healthcare professionals. We found that severe cognitive impairment was negatively associated with concurrently using ≥6 medications, compared with mild or moderate cognitive impairment. A similar trend was noted in patients who used ≥8 and ≥10 medications. The results showed that the OR for each polypharmacy medication decreased from mild to severe cognitive impairment, and the total number of medications decreased with worsening cognitive function severity. Moderate DASC-21 severity is associated with impairments in remote memory, location orientation, social judgement, or physical ADLs [29]. The presence of moderate dementia and polypharmacy may prompt the goals of care discussion regarding the relative benefits of individual medications. This period is likely to be characterised by moderate to severe executive dysfunction and difficulty maintaining medication adherence, and may be the starting point for medication adjustment. Furthermore, severely impaired patients have impairments in remote memory, location awareness, social judgement, and physical ADLs, which may require considering treatment with end-of-life care in mind. Current or anticipated side effects, inappropriate polypharmacy, progression of frailty and cognitive and physical dysfunction, and shorter life expectancy as triggers for prescription discontinuation or drug adjustment have been referred to in various references [32,33,34], supporting the present study.

In the DASC-21 severity classifications, BZ, PPI, and statin use was significantly lower in severe disease than in normal and mild disease, however, this trend was not observed for STOPP-J and antithrombotic drugs. The use of antithrombotic medications to prevent cerebral infarction can have serious outcomes if discontinued. Previous studies have also demonstrated that these medications cannot be easily discontinued, even in the presence of cognitive decline or deterioration in ADLs [35], which is in line with our findings. In addition, STOPP-J contains many antipsychotics, which may increase the likelihood of prescribing due to delirium or altered consciousness associated with the progression of frailty. Reportedly, the prescribing of antipsychotics increases toward the end of life [36], which is also in line with our findings. Furthermore, it has been reported that statins should not be discontinued, even in patients with advanced severe dementia, because of the prognostic value of coronary artery disease and other conditions [37]. On the other hand, in a report of attempted statin discontinuation in patients with poor prognosis for life, discontinuation was safe [38]. In the present study, the number of medications was reduced in patients with severe disease on the DASC-21. This may be attributed to the fact that the DASC-21 reflects cognitive as well as life functions, thus identifying patients with more severe disease than other scales, resulting in a reduction of statin use as well. It is also reasonable to assume that the use of BZ was discontinued because of the high risk of delirium and falls, and concerns regarding adverse events and cognitive dysfunction associated with worsening ADLs [39]. In older patients with atrial fibrillation, the DASC-21 severity classification may be used as a discussion tool to initiate medication adjustments, including medication reductions, especially for moderate to severe cases. To our knowledge, this is the first study to report on the clinical assessment of cognitive impairment to determine the association between cognitive impairment severity and the number of medications in older patients with atrial fibrillation. There has been no consensus on the various factors that lead to initiating medication adjustments. While studies using the CGA and other methods have been reported [23], no evaluation method solely focuses on medication adjustment, and much is left to the healthcare professional’s judgement. It is also important to consider medication adjustments when changes in physical function occur. In a retrospective study conducted in the UK among hypertensive patients aged >80 years, there was a higher rate of non-adherence to antihypertensive medication in the 5 years before death among non-survivors than among survivors [40]. Patients with severe cognitive impairment, judged using the DASC-21, were frail, and based on the present results, medication adjustment at that point appeared to be a common clinical response. However, the number of medications administered reportedly did not decrease in patients with heart failure, even if they showed functional impairment and other factors, such as the underlying disease [41]. As previously mentioned, moderate severity of DASC-21 may be associated with impairments in remote memory, place orientation, social judgment, or physical ADLs, [29]; however, its association with a decreased number of medications remains unknown. As the severity of DASC-21 increases, instrumental, which are executive functions, and physical ADLs are more likely to decline, and inadequate social support, such as medication assistance, could be a risk for rehospitalisation [42,43]. Therefore, from the perspective of healthcare professionals, a decline in physical and executive functions may be the starting point for medication reconciliation. It is important to use CGAs, such as the DASC-21, to adjust medications while considering the disease significance and prioritisation of medications from a geriatric viewpoint.

Antithrombotic medication use was associated with polypharmacy, even in cases in which polypharmacy continued. In addition, there was no decrease in antithrombotic medication use over the cognitive severity classification. This suggests that antithrombotic medications may be preferentially continued despite worse cognitive status using the DASC-21, reflecting goals of care considerations. Frail patients were reported as less likely to be prescribed direct oral anticoagulants than cognitively intact patients [44]; however, antithrombotic medications were continued in terminally ill patients with severe dementia despite them showing a variety of coexisting functional impairments [35]. Non-adherence to antithrombotic medication was associated with cognitive decline (as judged using the MMSE), and it was not linked to ADL or IADL impairment [45]. Therefore, it is important to use antithrombotic medications safely in frail patients. There is no doubt that eliminating polypharmacy, including the adjustment of inappropriate medications, is vital to reduce the risk of adverse events and interactions in frail patients with atrial fibrillation. In this context, when a patient is unable to take their medication, advance care planning (ACP) should be agreed upon with the patient and family.

This study has several limitations. Due to data construction, the number of medications in the inpatient setting was used in the analysis. Essentially, the number of medications for outpatients with stable disease is desirable for analysis. Since the Japanese healthcare system is still in the process of functional differentiation of hospital beds compared to other countries, patients are often treated in the hospital until their diseases stabilise, and the number of medications at discharge approximates the number of medications in the outpatient setting. Therefore, to minimise the impact of the number of medications, the number of medications on the day before discharge was extracted. We used DPC data recorded during insurance claims for this analysis, however, an important limitation is that insurance claims data may not capture all diseases present in patients. To address these, we used the CCI to account for comorbidities and further extracted and adjusted the number of contributing diseases to eliminate as many influences as possible. The breakdown of responders to the DASC-21 questionnaire in this study was 5 from unknown, 321 from the patients themselves, 21 from facility staff, and 844 (70.9%) from family members. The DASC-21 assessment showed that when family members responded, Cronbach’s alpha was higher, and the association with various dementia assessments (MMSE and CDR) was stronger [25]. The high response rate of family members in this study suggests that the DASC-21 questionnaire is more reliable than that intended in the original paper. However, it is unclear whether the family members who responded under the special circumstances of hospitalisation accurately understood the patient’s cognitive and life functions. Since this study was conducted at a single institution, the results of this study cannot be applied to other countries. Therefore, it is important to consider each country’s healthcare systems and demographics, and future studies examining this issue in multiple populations are warranted.

## 5. Conclusions

We found that severe cognitive impairment, as judged using the DASC-21, was associated with decreased polypharmacy in patients with atrial fibrillation. On the other hand, antithrombotic medications continued to be used in these patients regardless of DASC-21 severity. This suggests that there may be some risk/benefit-related ACP, depending on the class of medications. We believe that the DASC-21-based evaluation of cognition among patients with atrial fibrillation may allow for medication adjustment in clinical practice, and that healthcare professionals can maximise patient benefits.

## Figures and Tables

**Table 1:** Characteristics of the study population.

	All		≤5 Medications		>6 Medications		*p*-Value
	*n* = 1191		*n* = 267		*n* = 924		
Sex, male, *n* (%)	584	49.0%	142	53.2%	442	47.8%	0.1236
Age, years, *n* (%)							0.0135 *
≤74	186	15.6%	55	20.6%	131	14.2%	
75–84	477	40.1%	91	34.1%	386	41.8%	
≥85	528	44.3%	121	45.3%	407	44.1%	
BMI, kg/m^2^, *n* (%)							0.0280 *
<18.5	197	16.5%	55	20.6%	142	15.4%	
≥18.5 to <25	716	60.1%	163	61.1%	553	59.9%	
≥25	278	23.3%	49	18.4%	229	24.8%	
Length of hospital stay, days, *n* (%)							0.4102
≤7	340	28.6%	82	30.7%	258	27.9%	
8 to 14	273	22.9%	67	25.1%	206	22.3%	
15 to 25	281	23.6%	60	22.5%	221	23.9%	
≥26	297	24.9%	58	21.7%	239	25.9%	
Hospitalisation pathway, *n* (%)							0.1408
Household	1060	89.0%	231	86.5%	829	89.7%	
Transfer	131	11.0%	36	13.5%	95	10.3%	
Emergency hospitalisation, *n* (%)	738	62.0%	177	66.3%	561	60.7%	0.0982
Discharge destination, *n* (%)							0.0013 *
Household	868	72.9%	174	65.2%	694	75.1%	
Transfer	323	27.1%	93	34.8%	230	24.9%	
Patient co-payment rate, *n* (%)							0.0194 *
0%	64	5.4%	12	4.5%	52	5.6%	
10%	930	78.1%	196	73.4%	734	79.4%	
30%	197	16.5%	59	22.1%	138	14.9%	
Charlson Comorbidity Index, *n* (%)							0.0003 *
0	120	10.1%	37	13.9%	83	9.0%	
1 or 2	686	57.6%	169	63.3%	517	56.0%	
≥3	385	32.3%	61	22.8%	324	35.1%	
Comorbidities, *n* (%)							
Connective tissue disease/rheumatic disease	22	1.9%	4	4.8%	18	3.7%	0.6306
Peptic ulcer disease	97	8.1%	17	20.5%	80	16.6%	0.2280
Diabetes without complications	279	23.4%	45	54.2%	234	48.5%	0.004 *
Diabetes with complications	77	6.5%	4	4.8%	73	15.1%	0.0002 *
Renal disease	90	7.6%	13	15.7%	77	16.0%	0.0592
Concomitant medication, *n* (%)							
Antithrombotic	894	75.1%	174	65.2%	720	77.9%	<0.0001 *
Benzodiazepines	152	12.8%	10	3.7%	142	15.4%	<0.0001 *
STOPP-J **	167	14.0%	15	5.6%	152	16.5%	<0.0001 *
Proton pump inhibitor	799	67.1%	107	40.1%	692	74.9%	<0.0001 *
Statins	410	34.4%	35	13.1%	375	40.6%	<0.0001 *
Number of medications, median (IQR)	8.0	(6–11)	4.0	(3–5)	9.0	(8–12)	<0.0001 *
DASC-21 total scores, median (IQR)	28.0	(23–45)	28.0	(22–51)	28.5	(23–44)	0.8235
DASC-21 dementia severity 4 classification							0.0005 *
Normal	649	54.5%	146	54.7%	503	54.4%	
Mild	158	13.3%	24	9.0%	134	14.5%	
Moderate	351	29.5%	81	30.3%	270	29.2%	
Severe	33	2.8%	16	6.0%	17	1.8%	

* *p* < 0.05, ** STOPP-J (except for benzodiazepine medications); statins: HMG-CoA reductase inhibitors; BMI: body mass index; STOPP-J: Screening Tool for Older Person’s Appropriate Prescriptions for Japanese; DASC-21: Dementia Assessment Sheet for Community-based Integrated Care System 21-Items; and IQR: interquartile range.

**Table 2:** Association between cognitive impairment severity evaluated using the DASC-21 and polypharmacy.

Variables	>6 Medications		>8 Medications		>10 Medications	
	aOR (95% CI)	*p*-Value	aOR (95% CI)	*p*-Value	aOR (95% CI)	*p*-Value
Sex, male	0.82 (0.59–1.13)	0.219	0.82 (0.62–1.07)	0.147	1.02 (0.78–1.34)	0.860
Age, years, ref ≤ 74						
75–84	1.87 (1.20–2.91)	0.006	1.49 (1.01–2.20)	0.047	1.47 (0.99–2.19)	0.057
≥85	2.08 (1.28–3.37)	0.003	1.42 (0.93–2.16)	0.105	1.55 (1.01–2.40)	0.045
BMI, kg/m^2^, ref < 18.5						
18.5–25	1.11 (0.73–1.68)	0.624	1.03 (0.71–1.49)	0.860	1.18 (0.80–1.74)	0.398
>25	1.45 (0.86–2.47)	0.167	1.33 (0.85–2.08)	0.216	1.38 (0.88–2.18)	0.161
Length of hospitalisation, days						
≥ 26	1.95 (1.29–2.94)	0.002	1.80 (1.28–2.53)	<0.001	1.69 (1.21–2.37)	0.002
Hospitalisation pathway						
Transfer	1.50 (0.85–2.65)	0.165	1.47 (0.90–2.40)	0.120	1.25 (0.75–2.07)	0.388
Emergency hospitalisation	0.81 (0.56–1.16)	0.248	0.85 (0.62–1.15)	0.285	0.92 (0.68–1.25)	0.603
Discharge destination						
Transfer	0.50 (0.32–0.78)	0.002	0.64 (0.43–0.93)	0.020	0.63 (0.43–0.93)	0.020
CCI, ref = 0						
1–2	1.19 (0.73–1.94)	0.479	1.18 (0.76–1.84)	0.461	1.35 (0.83–2.19)	0.225
≥3	1.76 (0.98–3.15)	0.058	1.60 (0.96–2.66)	0.074	1.63 (0.95–2.81)	0.077
Comorbidities						
Connective tissue disease/rheumatic disease	0.96 (0.30–3.09)	0.951	1.66 (0.60–4.58)	0.328	3.16 (1.23–8.08)	0.017
Peptic ulcer disease	1.14 (0.62–2.09)	0.679	1.21 (0.74–1.99)	0.439	1.41 (0.89–2.26)	0.146
Diabetes without complications	1.48 (0.99–2.22)	0.058	1.57 (1.13–2.18)	0.007	1.28 (0.94–1.76)	0.122
Diabetes with complications	4.46 (1.50–13.2)	0.007	3.60 (1.78–7.24)	<0.001	2.90 (1.68–5.03)	<0.001
Renal disease	1.78 (0.87–3.64)	0.113	1.91 (1.08–3.36)	0.026	1.56 (0.93–2.62)	0.091
DASC-21 dementia severity 4 classification, ref = severe						
Normal	2.04 (0.84–4.94)	0.116	3.13 (1.23–8.01)	0.017	3.76 (1.04–13.5)	0.043
Mild	3.33 (1.29–8.57)	0.013	4.16 (1.58–10.9)	0.004	4.26 (1.16–15.6)	0.029
Moderate	2.46 (1.06–5.72)	0.036	3.68 (1.48–9.18)	0.005	3.95 (1.11–14.0)	0.033
Concomitant medication						
Antithrombotic	2.11 (1.51–2.95)	<0.001	2.42 (1.79–3.27)	<0.001	2.01 (1.46–2.77)	<0.001
BZs	5.25 (2.65–10.4)	<0.001	4.42 (2.77–7.05)	<0.001	4.04 (2.74–5.96)	<0.001
STOPP-J	4.26 (2.36–7.69)	<0.001	2.29 (1.53–3.42)	<0.001	1.90 (1.31–2.75)	0.002
Statins	4.36 (2.91–6.52)	<0.001	3.07 (2.29–4.12)	<0.001	2.49 (1.89–3.28)	<0.001

DASC-21: Dementia Assessment Sheet for Community-based Integrated Care System 21-Items; aOR: adjusted odds ratio; CI: confidence interval; ref: reference; BMI: body mass index; CCI: Charlson Comorbidity Index; BZs: benzodiazepines; and statins: HMG-CoA reductase inhibitors. DASC-21: Dementia Assessment Sheet for Community-based Integrated Care System 21-Items and STOPP-J: Screening Tool for Older Person’s Appropriate Prescriptions for Japanese. Multivariate logistic regression analysis was adjusted for the following covariates: sex, age, BMI, length of hospitalisation, hospitalisation pathway, emergency medical admissions, discharge destination, CCI, connective tissue disease/rheumatic disease, peptic ulcer disease, diabetes without complications, diabetes with complications, renal disease, antithrombotics, BZs, STOPP-J medications, and statins.

**Table 3:** Multiple comparison analysis of the number of medications per the severity of cognitive impairment based on the DASC-21.

	Number of Medications (per 100 Patients), Mean ± SD				Multiple Comparison Analysis *, *p*-Value		
DASC-21 Classification	Normal	Mild	Moderate	Severe	Normal	Mild	Moderate
Antithrombotics	76.7 ± 42.3	73.4 ± 44.3	73.2 ± 44.3	69.6 ± 46.7	0.501	0.855	0.855
Benzodiazepines	18.0 ± 44.4	15.8 ± 41.5	7.69 ± 28.7	0	0.026	0.041	0.179
STOPP-J medications	8.9 ± 31.6	19.6 ± 41.4	21.7 ± 43.9	12.1 ± 33.1	0.577	0.491	0.356
Proton pump inhibitors	67.5 ± 46.9	68.4 ± 46.7	67.8 ± 46.8	45.5 ± 50.6	0.017	0.023	0.018
Statins	40.5 ± 49.1	34.8 ± 47.8	25.1 ± 43.4	12.1 ± 33.1	0.002	0.019	0.154

* Steel’s multiple comparison tests (reference: severe).

## Data Availability

The datasets generated during and/or analysed during the current study are not publicly available to protect the participants’ privacy.

## Supplementary Materials

The following supporting information can be downloaded at: https://www.mdpi.com/article/10.3390/geriatrics9010015/s1, File S1: Generic name and ATC code for each drug type.
