# Supplementary material for: Association between Cognitive Impairment Severity and Polypharmacy in Older Patients with Atrial Fibrillation: A Retrospective Study Using Inpatient Data from a Specialised Geriatric Hospital
Source: Geriatrics (Basel). 2024 Jan 24;9(1):15. doi: 10.3390/geriatrics9010015 (PMC10887641; doi:10.3390/geriatrics9010015)
Supplement: Supplementary file 1 [file geriatrics-09-00015-s001.zip › geriatrics-2673086-supplementary.pdf]

Supplemental File S1

Generic name and ATC code for each drug type

List of generic names and drug codes for drugs on the list of drugs requiring particular caution

*ATC code does not exist.

・STOPP-J-covered drugs

Classification	Drug (class or generic name)	English generic name	NHI drug price Listed drug code	ATC code
Antipsychotics	Typical antipsychotics	Oxypertine	1179011	N05AE01
		Clomacramine hydrochloride	1179030	N05AX
		Chlorpromazine phenolphthalinate	1171005	N05AA01
		Chlorpromazine hydrochloride	1171001	N05AA01
		Spiperone	1179015	N05AD
		Sulthiame hydrochloride	1179032	N05AL02
		Zotepine	1179024	N05AX11
		Tiapride hydrochloride	1190004	N05AL03
		Timiperone	1179026	N05AD
		Nemonapride	1179036	N05AL
		Haloperidol	1179020	N05AD01
		Pipamperone (fluoropipamide hydrochloride)	1179006	N05AD05
		Pimozide	1179022	N05AG02
		Fluphenazine maleate	1172009	N05AB02
		Prochlorperazine maleate	1172010	N05AB04
		Propisephrine	1172005	N05AC01
		Bromperidol	1179028	N05AD06
		Perphenazine	1172006<br>1172007	N05AB03
		Perphenazine fendizoate	1172004	N05AB03
		Perphenazine maleate	1172013	N05AB03
		Mosapramine hydrochloride	1179035	N05AX10
		Levomepromazine maleate	1172014	N05AA02
Antipsychotics	Atypical antipsychotics	Asenapine maleate	1179056	N05AH05
		Aripiprazole	1179045	N05AX12
		Olanzapine	1179044	N05AH03
		Quetiapine fumarate	1179042	N05AH04

		Clozapine	1179049	N05AH02
		Paliperidone	1179053	N05AX13
		Blonanserin	1179048	N05AX
		Perospirone hydrochloride hydrate	1179043	N05AX
		Risperidone	1179038	N05AX08
Antidepressants	Tricyclic antidepressants	Amitriptyline hydrochloride	1179002	N06AA09
		Amoxapine	1179001	N06AA17
		Imipramine hydrochloride	1174006	N06AA02
		Clomipramine hydrochloride	1174002	N06AA04
		Dosulepin hydrochloride	1179027	N06AA16
		Trimipramine maleate	1174005	N06AA06
		Nortriptyline hydrochloride	1179004	N06AA10
		Lofepramine hydrochloride	1174004	N06AA07
Antidepressants	Selective serotonin receptor antagonists	Escitalopram oxalate	1179054	N06AB10
		Paroxetine hydrochloride hemihydrate	1179041	N06AB05
		Flupentixol maleate	1179039	N06AB08
		Sertraline hydrochloride	1179046	N06AB06
Sulpiride	Sulpiride	Sulpiride	1179016<br>2329009	N05AL01
Antiparkinsonian drugs	Antiparkinsonian drugs (anticholinergics)	Trihexyphenidyl hydrochloride	1169001<br>1169002	N04AA01
		Biperiden hydrochloride	1162001	N04AA02
		Pyroheptine hydrochloride	1169003	N04AA
		Prophephenamine hibenzate	1163002	N04AA05
		Prophephenamine hydrochloride	1163001	N04AA05
		Promethazine hydrochloride	4413002	R06AD02
		Promethazine hibenzate	4413002	R06AD02
		Promethazine methylenedisalicylate	4413002	R06AD02
		Methacholine chloride hemihydrate	1169004	N04AA10
Systemic corticosteroids	Oral steroids	Cortisone acetate	2452001	H02AB10
		Dexamethasone	2454002	H02AB02
		Triamcinolone	2454003	H02AB08
		Hydrocortisone	2452002	H02AB09
		Prednisolone	2456001<br>2456002	H02AB06
		Betamethasone	2454004	H02AB01
		Methylprednisolone	2456003	H02AB04
		Betamethasone<br>D-chlorpheniramine maleate	2459100	*
Antithrombotic agents (antiplatelet agents and anticoagulants)	Antiplatelet agents	Aspirin	3399007	B01AC06
		Clopidogrel sulfate	3399008	B01AC04
		Cilostazol	3399002	B01AC23
		Ticagrelor	3399011	B01AC24

		Ticlopidine hydrochloride	3399001	B01AC05
		Prasugrel hydrochloride	3399009	B01AC22
		Aspirin<br>Dihydroxyaluminum aminoacetate<br>Magnesium carbonate	3399100	*
		Aspirin<br>Clopidogrel bisulfate	3399101	*
		Aspirin<br>Lansoprazole	3399102	*
	Aspirin	Aspirin	1143001<br>3399007	N02BA01<br>B01AC06
		Aspirin<br>Dihydroxyaluminum aminoacetate<br>Magnesium carbonate	1143010<br>3399100	*
		Aspirin<br>Clopidogrel bisulfate	3399101	*
		Aspirin<br>Lansoprazole	3399102	*
	Combination of multiple antithrombotic drugs (antiplatelet agents and anticoagulants)	Aspirin	3399007	B01AC06
		Clopidogrel sulfate	3399008	B01AC04
		Cilostazol	3399002	B01AC23
Antithrombotic agents (antiplatelet agents and anticoagulants)	Combination of multiple antithrombotic drugs (antiplatelet agents and anticoagulants)	Ticagrelor	3399011	B01AC24
		Ticlopizine hydrochloride	3399001	B01AC05
		Prasugrel hydrochloride	3399009	B01AC22
		Aspirin<br>Dihydroxyaluminum aminoacetate<br>Magnesium carbonate	3399100	*
		Aspirin<br>Clopidogrel bisulfate	3399101	*
		Aspirin<br>Lansoprazole	3399102	*
		Apixaban	3339004	B01AF02
		Edoxaban tosylate hydrate	3339002	B01AF03
		Dabigatran etexilate mesylate	3339001	B01AE07
		Rivaroxaban	3339003	B01AF01
		Warfarin potassium	3332001	B01AA03
Cardiac glycosides	Digoxin	Digoxin	2113003<br>2113004	C01AA05
Diuretic	Loop diuretics	Azosemide	2139008	C03CA
		Torasemide	2139009	C03CA04
		Piretanide	2139007	C03CA03
		Bumetanide	2139004	C03CA02
		Furosemide	2139005	C03CA01
	Aldosterone antagonists	Eplerenone	2149045	C03DA04

		Spironolactone	2133001	C03DA01
Beta-blocking agents	Non-selective beta-blockers	Alprenolol hydrochloride	2123002	C07AA01
		Arotinolol hydrochloride	2123014	C07AA
		Carteolol hydrochloride	2123005<br>2149025	C07AA15
		Nadolol	2123015	C07AA12
		Nipradilol	2149021	C07AA
		Pindolol	2123009<br>2149011	C07AA03
		Bupentolol hydrochloride	2123006	C07AA
		Propranolol hydrochloride	2123008<br>2149014	C07AA05
Antihypertensives	Receptor subtype nonselective alpha 1 receptor blockers	Urapidil	2149020	C02CA06
		Terazosin hydrochloride hydrate	2149023	G04CA03
		Doxazosin mesylate	2149026	C02CA04
		Bunazosin hydrochloride	2149015	C02CA
		Prazosin hydrochloride	2149002	C02CA01
Antihistamines	1st-generation H1 receptor antagonists	D-chlorpheniramine maleate	4419002	R06AB04
		Alimemazine tartrate	4413003	R06AD01
		Clemastine fumarate	4419008	R06AA04
		Chlorpheniramine maleate	4419001<br>4419003	R06AB04
		Diphenhydramine	4411001	R06AA02
		Cyproheptadine hydrochloride	4419005	R06AX02
		Hydroxyzine pamoate	1179019	N05BB01
		Hydroxyzine hydrochloride	1179005	N05BB01
		Promethazine hydrochloride	4413002	R06AD02<br>D04AA10
		Promethazine hibenzate	4413002	R06AD02<br>D04AA10
		Promethazine methylenedisalicylate	4413002	R06AD02<br>D04AA10
		Homochlorcyclizine hydrochloride	4419006	R06AE
		Betamethasone<br>D-chlorpheniramine maleate	2459100	*
H2-receptor antagonists	H2-receptor antagonists	Cimetidine	2325001	A02BA01
		Nizatidine	2325005	A02BA04
		Famotidine	2325003	A02BA03
		Ranitidine hydrochloride	2325002	A02BA02
		Lafutidine	2325006	A02BA08
		Roxatidine acetate hydrochloride	2325004	A02BA06
Antiemetic drugs	Antiemetic drugs	Prochlorperazine maleate	1172010	N05AB04
		Promethazine hydrochloride	4413002	R06AD02<br>D04AA10

		Promethazine hibenzate	4413002	R06AD02<br>D04AA10
		Promethazine methylenedisalicylate	4413002	R06AD02<br>D04AA10
		Metoclopramide	2399004	A03FA01
Laxatives	Magnesium oxide	Magnesium oxide	2344002<br>2344009	A02AA02<br>A06AD02
Antidiabetic drugs	Sulfonylurea drugs	Acetohexamide	3961001	A10BB31
		Gliclazide	3961007	A10BB09
		Glipizide	3961002	A10BB
		Glibenclamide	3961003	A10BB01
		Glimepiride	3961008	A10BB12
		Chlorpropamide	3961004	A10BB02
		Tolbutamide	3961006	A10BB03
		Glimepiride pioglitazone hydrochloride	3969101	A10BD06
	Biguanides	Buformin hydrochloride	3962001	A10BA03
		Metformin hydrochloride	3962002	A10BA02
		Metformin hydrochloride alogliptin benzoate	3969105	A10BD13
		Metformin hydrochloride vildagliptin hydrochloride	3969104	A10BD08
		Metformin hydrochloride pioglitazone hydrochloride	3969100	A10BD05
	Thiazolidinediones	Pioglitazone hydrochloride	3969007	A10BG03
		Pioglitazone hydrochloride alogliptin benzoate	3969103	A10BD09
		Pioglitazone hydrochloride glimepiride	3969101	A10BD06
		Metformin hydrochloride pioglitazone hydrochloride	3969100	A10BD05
	Alpha-glucosidase inhibitors	Acarbose	3969003	A10BF01
		Voglibose	3969004	A10BF03
		Miglitol	3969009	A10BF02
		Mitiglinide calcium hydrate voglibose	3969102	*
	SGLT2 inhibitors	Ipragliflozin L-proline	3969018	A10BK
		Empagliflozin	3969023	A10BK03
		Canagliflozin hydrate	3969022	A10BK02
		Dapagliflozin propylene glycol hydrate	3969019	A10BK01
		Tofogliflozin hydrate	3969021	A10BK
		Luseogliflozin hydrate	3969020	A10BK
Drugs for overactive bladder	Oxybutynin (oral)	Oxybutynin hydrochloride	2590005	G04BD04
	Muscarinic receptor antagonists	Imidafenacin	2590013	G04BD
		Solifenacin succinate	2590011	G04BD08
		Tolterodine tartrate	2590012	G04BD07
		Fesoterodine fumarate	2590015	G04BD11
		Propiverine hydrochloride	2590007	G04BD06
NSAIDs	NSAIDs	Acemetacin	1145003	M01AB11
		Ampiroxicam	1149030	M01AC
		Amfenac sodium hydrate	1147006	M01AB

Ibuprofen	1149001	M01AE01
Indomethacin	1145001<br>1145002	M01AB01
Indometacin farnesil	1145005	M01AB01
Etodolac	1149032	M01AB08
Emorphazone	1148004	N02BG
Oxaprozin	1149026	M01AE12
Zaltoprofen	1149029	M01AE
Diclofenac sodium	1147002	M01AB05
Sulindac	1149015	M01AB02
Tiaprofenic acid	1149025	M01AE11
Tiaramide hydrochloride	1148001	N02BG
Nabumetone	1149027	M01AX01
Naproxen	1149007	M01AE02
Piroxicam	1149017	M01AC01
Bucloxic acid	1149009	M01AX
Pranoprofen	1149010	S01BC09
Flufenamic acid aluminum	1141004	M01AG03
Flurbiprofen	1149011	M01AE09
Proglumetacin maleate	1145004	M01AB14
Mefenamic acid	1141005	M01AG01
Meloxicam	1149035	M01AC06
Mofezolac	1149033	M01AX
Loxoprofen sodium hydrate	1149019	M01AE
Lornoxicam	1149036	M01AC05

<Notes for patients eligible according to STOPP-J>

When each medication on this list is used in an ‘eligible patient population’, it should be administered with ‘special caution’; however, since the intent of the administration is unknown, the eligibility of patients receiving each of these medications according to STOPP-J should be considered.

・Benzodiazepines

Classification	Drug (class or generic name)	English generic name	NHI drug price<br>Listed drug code	ATC code
Sleep-inducing tranquilizers	Benzodiazepines	Alprazolam	1124023	N05BA12
		Estazolam	1124001	N05CD04
		Etizolam	1179025	N05BA19
		Oxazolam	1124013	N05BA
		Quazepam	1124030	N05CD10
		Cloxazolam	1124014	N05BA22
		Clotiazepam	1179012	N05BA21

		Potassium clorazepate	1124015	N05BA05
		Clonazepam	1139003	N03AE01
		Clobazam	-	N05BA09
		Chlordiazepoxide	1124028	N05BA02
		Diazepam	1124017	N05BA01
		Tofisopam	1124026	N05BA23
		Triazolam	1124007	N05CD05
		Nitrazepam	1124003	N05CD02
		Nimetazepam	1124004	N05BA
		Haloxazolam	1124005	N05CD
		Fludiazepam	1124019	N05BA17
		Flutazolam	1124024	N05BA
		Flutoprazepam	1124027	N05BA
		Flunitrazepam	1124008	N05CD03
		Flurazepam hydrochloride	1124002	N05CD01
		Brotizolam	1124009	N05CD09
		Brotizolam	1124020	N05BA08
		Mexazolam	1124025	N05BA
		Medazepam	1124021	N05BA03
		Midazolam	1139700	N05CD08
		Lormetazepam hydrochloride hydrate	1129006	N05CD
		Ethyl loflazepate	1124029	N05BA18
		Lorazepam	1124022	N05BA06
		Lormetazepam	1124010	N05CD06
	Non-benzodiazepines	Eszopiclone	1129010	N05CF04
		Zopiclone	1129007	N05CF01
		Zolpidem	1129009	N05CF02

・Proton pump inhibitors

Classification	Drug (class or generic name)	English generic name	NHI drug price Listed drug code	ATC code
Proton pump inhibitors	Proton pump inhibitors	Omeprazole	2329022	A02BC01
		Lansoprazole	2329023	A02BC03
		Rabeprazole sodium	2329028	A02BC04
		Esomeprazole magnesium hydrate	2329029	A02BC05
		Vonoprazan fumarate	2329030	A02BC08
		Aspirin-Lansoprazole combination	3399102	*
		Aspirin-Vonoprazan fumarate combination	3399103	*

・HMG CoA reductase inhibitors

Classification	Drug (class or generic name)	English generic name	NHI drug price Listed drug code	ATC code
HMG CoA reductase inhibitors	HMG CoA reductase inhibitors	Pravastatin sodium	2189010	C10AA03
		Simvastatin	2189011	C10AA01
		Fluvastatin sodium	2189012	C10AA04
		Atorvastatin calcium hydrate	2189015	C10AA05
		Pitavastatin calcium	2189016	C10AA08
		Rosuvastatin calcium	2189017	C10AA07
		Ezetimibe-Atorvastatin calcium hydrate	2189101	C10BA05
		Ezetimibe-Rosuvastatin calcium	2189102	C10BA06
		Pitavastatin calcium hydrate-Ezetimibe	2189103	*
		Amlodipine besylate-Atorvastatin calcium hydrate	2190101	C10BX03
		Amlodipine besylate-Atorvastatin calcium hydrate	2190102	C10BX03
		Amlodipine besylate-Atorvastatin calcium hydrate	2190103	C10BX03
		Amlodipine besylate-Atorvastatin calcium hydrate	2190104	C10BX03

・Antithrombotic agents

Classification	Drug (class or generic name)	English generic Name	NHI drug price Listed drug code	ATC code
Antithrombotic agents	Antithrombotic agents	Apixaban	3339004	B01AF02
		Edoxaban tosylate hydrate	3339002	B01AF03
		Dabigatran etexilate mesylate	3339001	B01AE07
		Rivaroxaban	3339003	B01AF01
		Warfarin potassium	3332001	B01AA03

Characteristics of the study population (each case of polypharmacy)

	All		≤5 medications		>6 medications		*P*-value	≤7 medications		>8 medications		*P*-value	≤9 medications		>10 medications		*P*-value
	n = 1193		n = 267		n = 924			n = 492		n = 699			n = 750		n = 441		
Sex, n (%)							0.1236*					0.106*					0.5292*
Male	584	49.0%	142	53.2%	442	47.8%		255	51.8%	329	47.1%		373	49.7%	211	47.9%	
Female	607	51.0%	125	46.8%	482	52.2%		237	48.2%	370	52.9%		377	50.3%	230	52.1%	

Age, years							0.0135*					0.0202*					0.0568*
≤74	186	15.6%	55	20.6%	131	14.2%		85	17.3%	101	14.5%		124	16.5%	62	14.1%	
75-84	477	40.1%	91	34.1%	386	41.8%		174	35.4%	303	43.4%		281	37.5%	196	44.4%	
≥85	528	44.3%	121	45.3%	407	44.1%		233	47.4%	295	42.2%		345	46.0%	183	41.5%	
BMI category classification, n (%)							0.0280*					0.0121*					0.0162*
<18.5	197	16.5%	55	20.6%	142	15.4%		94	19.1%	103	14.7%		138	18.4%	59	13.4%	
≥18.5 to <25	716	60.1%	163	61.1%	553	59.9%		302	61.4%	414	59.2%		453	60.4%	263	59.6%	
≥25	278	23.3%	49	18.4%	229	24.8%		96	19.5%	182	26.0%		159	21.2%	119	27.0%	
Length of hospital stay , days							0.4102*					0.3262*					0.3988*
Less than 7	340	28.6%	82	30.7%	258	27.9%		144	29.3%	196	28.0%		226	30.1%	114	25.8%	
8-14	273	22.9%	67	25.1%	206	22.3%		123	25.0%	150	21.5%		172	22.9%	101	22.9%	
15-25	281	23.6%	60	22.5%	221	23.9%		113	23.0%	168	24.0%		173	23.1%	108	24.5%	
>26	297	24.9%	58	21.7%	239	25.9%		112	22.8%	185	26.5%		179	23.9%	118	26.8%	
Hospitalisation pathway, n (%)							0.1408*					0.1381*					0.0273*
Household	1060	89.0%	231	86.5%	829	89.7%		430	87.4%	630	90.1%		656	87.5%	404	91.6%	
Transfer	131	11.0%	36	13.5%	95	10.3%		62	12.6%	69	9.9%		94	12.5%	37	8.4%	
Emergency hospitalisation, n (%)	738	62.0%	177	66.3%	561	60.7%	0.0982*	320	65.0%	418	59.8%	0.0666*	477	63.6%	261	59.2%	0.1295*
Discharge destination, n (%)							0.0013*					0.0028*					0.0009*
Household	868	72.9%	174	65.2%	694	75.1%		336	68.3%	532	76.1%		522	69.6%	346	78.5%	
Transfer	323	27.1%	93	34.8%	230	24.9%		156	31.7%	167	23.9%		228	30.4%	95	21.5%	
Patient co-payment rate							0.0194*					0.0042*					0.0045*
0%	64	5.4%	12	4.5%	52	5.6%		16	3.3%	48	6.9%		31	4.1%	33	7.5%	

10%	930	78.1%	196	73.4%	734	79.4%		381	77.4%	549	78.5%		580	77.3%	350	79.4%	
30%	197	16.5%	59	22.1%	138	14.9%		95	19.3%	102	14.6%		139	18.5%	58	13.1%	
Charlson Comorbidity Index, n (%)							0.0003*					<.0001*					<.0001*
0	120	10.1%	37	13.9%	83	9.0%		62	12.6%	58	8.3%		88	11.7%	32	7.3%	
1 or 2	686	57.6%	169	63.3%	517	56.0%		305	62.0%	381	54.5%		448	59.7%	238	54.0%	
≥3	385	32.3%	61	22.8%	324	35.1%		125	25.4%	260	37.2%		214	28.5%	171	38.8%	
Comorbidities, n (%)																	
Connective tissue disease-rheumatic disease	22	1.9%	4	4.8%	18	3.7%	0.6306*	6	3.6%	16	4.0%	0.1771*	8	1.1%	14	3.2%	0.0091*
Peptic ulcer disease	97	8.1%	17	20.5%	80	16.6%	0.2280*	33	19.9%	64	16.0%	0.1282*	52	6.9%	45	10.2%	0.0463*
Diabetes without complications	279	23.4%	45	54.2%	234	48.5%	0.004*	89	53.6%	190	47.6%	0.0003*	159	21.2%	120	27.2%	0.0180*
Diabetes with complications	77	6.5%	4	4.8%	73	15.1%	0.0002*	12	7.2%	65	16.3%	<.0001*	28	3.7%	49	11.1%	<.0001*
Renal disease	90	7.6%	13	15.7%	77	16.0%	0.0592*	26	15.7%	64	16.0%	0.0128*	47	6.3%	43	9.8%	0.0280*
Concomitant medication, n (%)																	
Antithrombotic agents	894	75.1%	174	65.2%	720	77.9%	<.0001*	327	66.5%	567	81.1%	<.0001*	531	70.8%	363	82.3%	<.0001*
Benzodiazepines	152	12.8%	10	3.7%	142	15.4%	<.0001*	27	5.5%	125	17.9%	<.0001*	56	7.5%	96	21.8%	<.0001*
STOPP-J***	167	14.0%	15	5.6%	152	16.5%	<.0001*	49	10.0%	118	16.9%	<.0001*	90	12.0%	77	17.5%	0.0088*
Proton pump inhibitors	799	67.1%	107	40.1%	692	74.9%	<.0001*	250	50.8%	549	78.5%	<.0001*	427	56.9%	372	84.4%	<.0001*
Statins	410	34.4%	35	13.1%	375	40.6%	<.0001*	99	20.1%	311	44.5%	<.0001*	197	26.3%	213	48.3%	<.0001*
Number of medications, median (IQR)							<.0001**					<.0001**					<.0001**
	8.0	(6-11)	4.0	(3-5)	9.0	(8-12)		5.0	(4-6)	10.0	(9-12)		6.0	(4-8)	12.0	(10-14)	
DASC-21 total scores, median (IQR)							0.8235**					0.6990**					0.2165**
	28.0	(23-45)	28.0	(22-51)	28.5	(23-44)		28.5	(22-50)	28.0	(23-43)		29.0	(23-48)	28.0	(23-41)	

DASC-21 dementia severity 4 classification							0.0005*					0.0003*					0.0036*
Normal	649	54.5%	146	54.7%	503	54.4%		262	53.3%	387	55.4%		397	52.9%	252	57.1%	
Mild	158	13.3%	24	9.0%	134	14.5%		56	11.4%	102	14.6%		94	12.5%	64	14.5%	
Moderate	351	29.5%	81	30.3%	270	29.2%		149	30.3%	202	28.9%		229	30.5%	122	27.7%	
Severe	33	2.8%	16	6.0%	17	1.8%		25	5.1%	8	1.1%		30	4.0%	3	0.7%	

*Chi-square test (Pearson test), **Mann-Whitney *U* test, ***STOPP-J (except benzodiazepines), Statins: HMG-CoA reductase inhibitors

ATC: Anatomical Therapeutic Classification, STOPP-J: Screening Tool for Older Person's Appropriate Prescriptions for Japanese, NHI: National Health Insurance, SGLT2: sodium glucose cotransporter 2, NSAIDs: nonsteroidal anti-inflammatory drugs, HMG CoA: 3-hydroxy-3-methylglutaryl-coenzyme A, BMI: body mass index, IQR: interquartile range, DASC-21: Dementia Assessment Sheet for Community-based Integrated Care System 21-Items
